# Microelements and biochemical biomarkers-based machine learning for predicting adverse pregnancy outcomes in Wilson’s disease: risk stratification by integrating hepatic fibrosis and cerebral function

**DOI:** 10.3389/fnut.2026.1768588

**Published:** 2026-02-17

**Authors:** Juan Wang, Qing-qing Ming, Yong-guang Shi, Yin Xu, Jun-cang Wu, Xu-en Yu, Xu Zhang

**Affiliations:** 1 Department of Neurology, Hefei Hospital Affiliated to Anhui Medical University (Hefei Second People’s Hospital), Department of Health Management Center, First Affiliated Hospital of Anhui Medical University, Hefei, China; 2 Institute of Neurology, Anhui University of Chinese Medicine, Department of Neurology, Affiliated Hospital of the Neurology Research Institute of Anhui University of Chinese Medicine, Hefei, China

**Keywords:** adverse pregnancy outcomes, generalized linear model, machine learning, microelements, uneventful pregnancy, Wilson’s disease

## Abstract

**Background:**

Pregnancy in female patients with Wilson’s disease (WD) raises significant gestational risks due to potential adverse pregnancy outcomes (APOs). This study developed machine learning (ML) algorithms based on microelement profiles and biochemical markers to identify APOs.

**Methods:**

Data on microelements (e.g., serum/urinary copper, iron), biochemical markers, and hepatic fibrosis were measured for all patients. Feature selection was performed using LASSO regression. Four ML models, including generalized linear model (GLM), deep learning (DL), random forest (RF), and gradient boosting machine (GBM), were developed and validated to distinguish between APOs and uneventful pregnancies (UP). Stratified analyses were conducted based on cerebral function (normal cerebral function *vs.* abnormal cerebral dysfunction) and hepatic fibrosis (with *vs.* without hepatic fibrosis).

**Results:**

114 patients with WD were enrolled, including 57 APO and 57 UP. The APO group exhibited a shorter disease duration, insufficient pre-pregnancy decoppering therapy, elevated levels of 24-h urinary copper and serum iron, and increased hepatic fibrosis biomarkers. Of the four ML models, the GLM had the highest accuracy (0.850) in the test set with excellent stability across training, test and validation sets, and no overfitting. RF and GBM had overfitting, while DL demonstrated poor generalization capability. Additionally, stratified analysis confirmed that the GLM showed strong robustness in most subgroups, whereas the GBM performed best performance in WD patients with cerebral dysfunction.

**Conclusion:**

Microelements imbalance and hepatic fibrosis are associated with the risk of APOs in WD patients. The GLM, except for WD patients with cerebral dysfunction, serves as a reliable and generalizable predictive tool for APOs.

## 1 Introduction

Wilson’s disease (WD) is a rare autosomal recessive disorder of copper metabolism caused by mutations in the ATP7B gene, leading to toxic copper accumulation in the brain and liver (1–3). Fertility issues in women of childbearing age with WD are often overlooked, and information on pregnancy outcomes in patients with WD remains scarce (4–6). Existing studies indicated a high incidence of miscarriage (or recurrent miscarriage) in untreated female WD patients, and some patients even experiencing recurrent pregnancy failures (4, 5, 7). With the availability of effective pharmacotherapy, such as chelating agents and zinc salts, most patients with WD can now survive to reproductive age and achieve successful pregnancies (5, 7). Consequently, the focus has shifted from achieving pregnancy to optimizing its outcomes.

Although most patients with WD are able to conceive successfully, consistent evidence indicated that these patients still have a higher residual risk of adverse pregnancy outcomes (APOs), such as preeclampsia, preterm birth, and fetal growth restriction, compared with the general population (4, 8), It suggests that merely being “receiving treatment” is insufficient. A key unresolved issue is that some patients with WD undergoing treatment experience can complete uneventful pregnancies (UP), while others develop APOs (9). The pathophysiological mechanisms underlying this increased risk are not fully understood but are generally associated with the complex interactions among copper homeostasis, oxidative stress, and endothelial dysfunction.

Pregnancy is a state of dynamic flux in microelements (10). In patients with WD, the occurrence of APOs is significantly associated with dysregulation of essential microelements, with copper playing a central and complex role (2, 11, 12). Excessive free copper readily participates in Fenton-like reactions, generating hydroxyl radicals and reactive oxygen species (ROS), thereby leading to oxidative damage to cellular lipids, proteins, and DNA (13, 14). Serum iron, frequently dysregulated in patients with WD, can act synergistically with serum copper to aggravate oxidative stress (14). Conversely, zinc, a physiological antagonist of copper and commonly used in the treatment of WD, may exert a protective effect and could be a key modulator of APO risk (14). The imbalances of these elements and their effects on placental health and fetal development are critical and multifaceted determinants; however, this delicate equilibrium is disrupted in patients with WD (14, 15). Therefore, although patients with WD can achieve successful pregnancies under treatment, their risk of APOs remains higher (4, 8). The precise relationship between fluctuations in microelements and APOs is currently unclear. Therefore, it is necessary to explore an accurate, sensitive, low-cost, and non-invasive detection method for the early identification of patients at potential risk of APOs.

In recent years, machine learning (ML) has been widely applied in biomedical research, disease diagnosis, and prediction, demonstrating its potential as an effective tool for early patient identification (16–18). However, no studies have yet investigated the role of ML models, especially microelements-based ML models, in differentiating and diagnosing APOs in patients with WD. This study characterizes the profiles of key microelements in patients with pregnant WD and uses four ML models to investigate their specific associations with APOs.

## 2 Materials and methods

### 2.1 Study design and participants

This retrospective study was conducted at the Institute of Neurology, Anhui University of Chinese Medicine, from January 2020 to July 2025. A total of 114 pregnant women with a confirmed diagnosis of WD (Leipzig score ≥4) were included. To ensure comparability and balance of data across different groups, patients were matched at a 1:1 ratio, resulting in 57 patients in the APO group and 57 patients in the UP group. The diagnosis of WD was independently established by two senior neurologists based on the Leipzig diagnostic criteria (2, 19), in accordance with a comprehensive assessment of clinical symptoms, laboratory tests, and genetic testing. This study was supported by the Clinical Hospital Research Ethics Committee of the Anhui University of Chinese Medicine (2024-SYSFYSY-17).

### 2.2 Inclusion and exclusion criteria

The inclusion criteria were as follows: (1) Pregnant women with a confirmed diagnosis of WD. (2) First pregnancy. (3) Provision of informed consent. (4) Aged ≥ 18 years old.

The exclusion criteria included: (1) Presence of other chronic liver diseases (e.g., viral hepatitis, autoimmune hepatitis). (2) History of severe renal insufficiency. (3) Not a first pregnancy. (4) Patients subjected to gynecological treatment. (5) Inability to comply with the study protocol.

### 2.3 Group definitions

Patients were divided into two groups according to strict criteria:

The UP group (*n* = 57): Patients delivered a live infant at ≥37 weeks of gestation, with neonatal birth weight between the 10th and 90th percentiles, and without hypertensive disorders of pregnancy, gestational diabetes mellitus, or clinical placental abruption.

APO group (*n* = 57): The composite outcome included the occurrence of one or more of the following: spontaneous abortion, missed abortion, stillbirth, and fetal developmental abnormalities.

### 2.4 Data collection and measurements

Demographic and clinical parameters were recorded, including age at first pregnancy, gender, disease duration, disease subtype, pregnancy outcomes, and whether standardized copper-elimination therapy had been administered prior to pregnancy. Additionally, Kayser–Fleischer rings, ultrasound detection of hepatic fibrosis, and magnetic resonance imaging (MRI) detection of cranial abnormalities were routinely assessed.

Microelements, including serum copper, ceruloplasmin, urine copper (the last recorded data before pregnancy), serum zinc, and serum iron, were measured. Serum biochemical indices, liver function parameters, and hepatic fibrosis markers, including white blood cell (WBC) count, red blood cell (RBC) count, platelet, alanine aminotransferase (ALT), aspartate aminotransferase (AST), *γ*-glutamyl transpeptidase (γ-GT), total protein, total bilirubin, hyaluronic acid, laminin, procollagen III, and collagen IV, were detected using standard automated biochemical analyzers (Hitachi) in the institution’s central laboratory.

### 2.5 Machine learning models

Seventy percent of the dataset was randomly allocated for training ML models, 15% for testing the models, and the remaining 15% for validation set. For WD patients without hepatic fibrosis, the sample size was extremely limited (*n* = 22). Therefore, 5-fold cross-validation was employed to evaluate the performance of ML models, and this approach was used as a sensitivity analysis. Four ML models, including deep learning (DL, fully connected deep neural network was used in this study), random forest (RF), gradient boosting machine (GBM), and generalized linear model (GLM), were utilized to distinguish between UP and APO in patients with WD. Detailed descriptions of ML model can be found in Supplementary materials, as previously published article (16).

### 2.6 Feature selection and preprocessing

Given the potential for multicollinearity among the independent variables, feature selection was performed prior to constructing ML models. Firstly, univariate analysis was conducted for all independent variables. Since variables were entered into the model individually at this stage, multicollinearity was not a concern. Secondly, variables with significant differences in the univariate analysis were further analyzed using least absolute shrinkage and selection operator (LASSO) regression. LASSO is widely used for screening variable due to its ability to handle multicollinearity. Subsequently, variables identified as significant by LASSO regression were further analyzed using multivariate analysis to determine independent risk factors associated with APOs. Finally, based on the independent risk factors, four ML models were constructed for the diagnosis and prediction of APOs.

### 2.7 Statistical analysis

Continuous variables were assessed for normal distribution using the Kolmogorov–Smirnov test. Data comparisons between the UP group and the APO group were conducted using the independent *T*-test (for normal distribution data) and the Mann–Whitney U test (for skewed distribution data), respectively. Categorical variables were described using sample sizes and percentages, and comparisons were analyzed using the chi-square test. Variables demonstrating significant difference (i.e., *p* < 0.05) in the univariate analysis were included in LASSO regression to identify independent risk factors for APOs. Subsequently, the prediction outcomes were visualized using a nomogram. Furthermore, four previously described ML models (16) were developed based on the identified significant variables. The predictive performance of all models was assessed using precision-recall (PR) curves, receiver operating characteristic (ROC) curves, and their respective area under the curve (AUC) values (i.e., AUCPR and AUCROC). As previously described (16), the changes of mean square error (MSE), root mean square error (RMSE), R square, as well as brier score, calibration-in-the-large, and calibration slope were calculated to evaluate model fitting performance. Stratified analyses were conducted for patients with hepatic fibrosis (with vs. without fibrosis) and cerebral function (abnormal vs. normal). All statistical analyses were performed using R software R software (XGBoost package, version 4.4.0), with *p* < 0.05 considered statistically significant.

## 3 Results

### 3.1 Cohort characteristics and pregnancy outcomes

**Figure 1:**
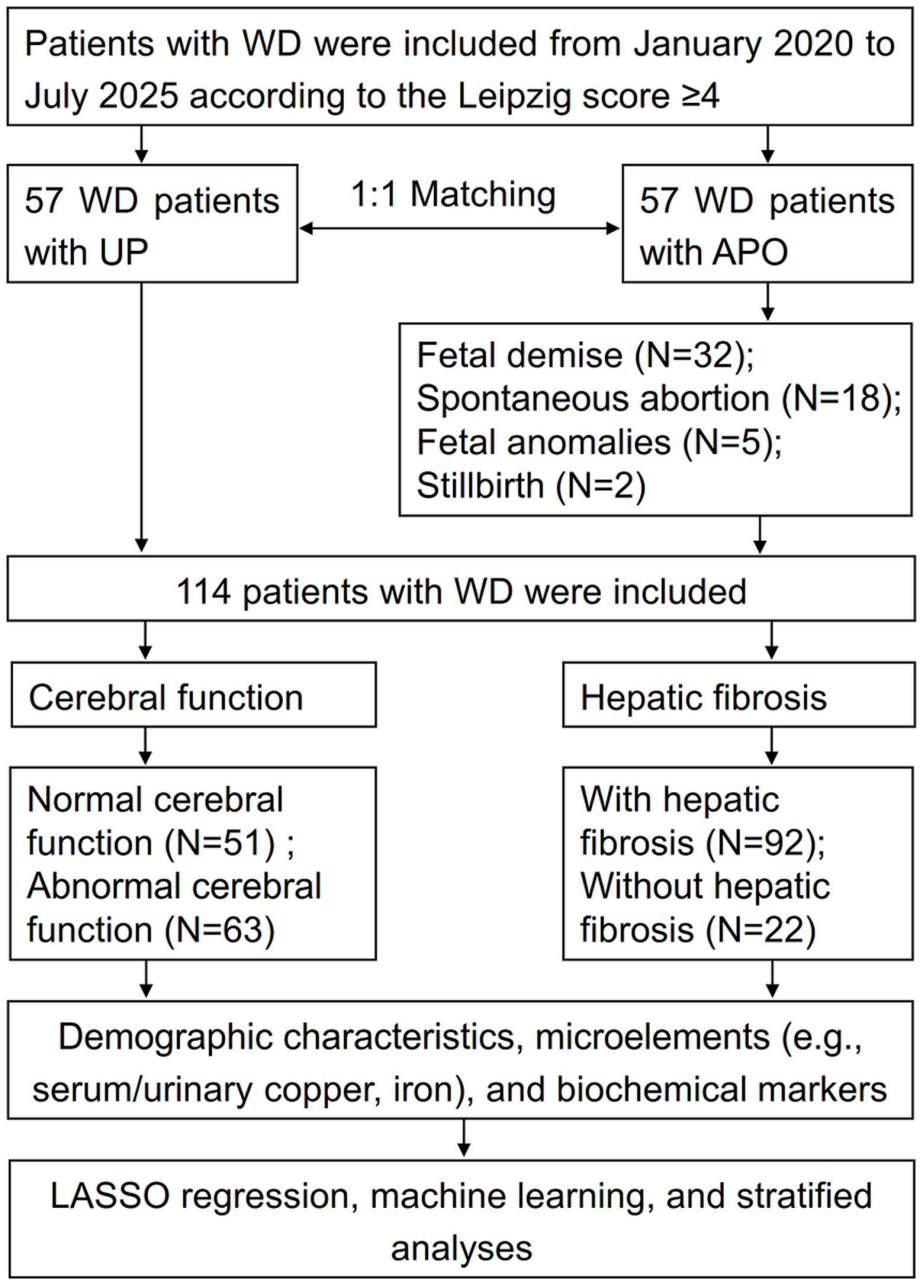
Study cohort flowchart.

Overall, a total of 114 patients with WD were included, comprising 57 WD patients with the UP and 57 WD patients with APO. Of 57 patients with APO, 32 cases involved fetal death, 18 cases involved spontaneous abortion, 5 cases involved fetal malformation, and 2 cases involved stillbirth (Figure 1; Table 1). No significant difference was observed in age at first pregnancy between UP and APO groups, but the disease duration in the APO group was shorter than that in the UP group (*Z* = −2.242, *p* < 0.001, Table 1). The APO group was predominantly observed in patients with the cerebral function of WD, whereas the UP group was mainly occurred in the hepatic fibrosis (*χ*^2^ = 23.392, *p* < 0.001, Table 1). Additionally, the APO group had not undergone adequate decoppering therapy prior to pregnancy (*χ*^2^ = 52.627, *p* < 0.001, Table 1) and exhibited pronounced clinical manifestations, including Kayser-Fleischer rings (*χ*^2^ = 28.977, *p* < 0.001, Table 1), hepatic fibrosis (*χ*^2^ = 5.632, *p* = 0.031, Table 1), and cerebral function (*χ*^2^ = 7.983, *p* = 0.008, Table 1).

**Table 1:** Characteristics of patients with WD.

Characteristics	UP (*n* = 57)	APO (*n* = 57)	*t*/*Z*/*χ*^2^	*p*
Fetal demise	NA	32	NA	NA
Spontaneous abortion	NA	18	NA	NA
Fetal anomalies	NA	5	NA	NA
Stillbirth	NA	2	NA	NA
Age at conception (years)	28.5 ± 3.7	29.5 ± 4.1	−1.343^a^	0.182
Disease duration (years)	12.0 (10.0, 18.0)	9.0 (4.0, 16.5)	−2.242^b^	0.025
Disease subtype (*n*, %)			23.392^c^	<0.001
Cerebral function	9 (15.8)	31 (54.4)		
Hepatic fibrosis	35 (61.4)	12 (21.1)		
Mixed type	13 (22.8)	14 (14.6)		
Pre_PT (*n*, %)			52.627^c^	<0.001
No	4 (7.0)	42 (73.7)		
Yes	53 (93.0)	15 (26.3)		
Kayser-Fleischer (*n*, %)			28.977^c^	<0.001
0	20 (35.1)	7 (12.3)		
1	10 (17.5)	2 (3.5)		
2	15 (26.3)	17 (29.8)		
3	11 (19.3)	13 (22.8)		
4	1 (1.8)	18 (31.6)		
Hepatic fibrosis (*n*, %)			5.632^c^	0.031
Without	16 (28.1)	6 (10.5)		
With	41 (71.9)	51 (89.5)		
Cerebral function (*n*, %)			7.983^c^	0.008
Normal	33 (57.9)	18 (31.6)		
Abnormal	24 (42.1)	39 (68.4)		

APO, adverse pregnancy outcome; UP, uneventful pregnancy; Pre_PT, pre-pregnancy treatment; NA, not applicable.

a *t* value.

b *Z* value.

c *χ*^2^ value.

### 3.2 Microelements and biochemical markers

In this study, the levels of microelements, including serum copper, ceruloplasmin, 24-h urinary copper, serum iron, and serum zinc, were measured (Table 2). The results showed that the levels of 24-h urinary copper (*Z* = −4.123, *p* < 0.001, Table 2), serum iron (*Z* = −2.596, *p* = 0.006, Table 2), platelet (*Z* = −3.160, *p* = 0.002, Table 2), ALT (*Z* = −2.693, *p* = 0.007, Table 2), AST (*Z* = −2.901, *p* = 0.004, Table 2), GGT (*Z* = −2.098, *p* = 0.038, Table 2), and total bilirubin (Z = −5.733, *p* < 0.001, Table 2) were elevated, whereas WBC (*t* = 2.038, *p* = 0.044, Table 2) and total protein (*Z* = 5.528, *p* < 0.001, Table 2) were decreased in the APO group compared to the UP group. Furthermore, hepatic fibrosis biomarkers, excepting for laminin (*Z* = −0.955, *p* = 0.340, Table 2), were significantly elevated in the APO group than in the UP group.

**Table 2:** Microelements and biochemical indicators in patients with WD.

Characteristics	UP (*n* = 57)	APO (*n* = 57)	*t*/*Z*	*p*
Microelements				
Serum copper (μmol/L)	1.9 (1.4, 3.2)	2.3 (1.5, 4.7)	−0.941^b^	0.347
Ceruloplasmin (mg/L)	42.6 (32.4, 62.3)	45.7 (35.9, 71.4)	−0.972^b^	0.331
Urine copper (ug/24 h)	604.6 (481.3, 684.0)	760.1 (595.1, 960.4)	−4.123^b^	<0.001
Serum zinc (μmol/L)	14.9 (11.8, 19.2)	15.2 (11.0, 22.6)	−0.388^b^	0.698
Serum iron (μmol/L)	10.2 (7.8, 14.4)	13.0 (9.8, 19.3)	−2.596^b^	0.009
Biochemical markers				
WBC (x10^9^/L)	5.1 ± 1.3	4.5 ± 1.7	2.038^a^	0.044
RBC (x10^12^/L)	4.2 ± 0.4	4.2 ± 0.5	0.471^a^	0.639
Platelet (x10^9^/L)	175.0 (140.1, 218.0)	141.0 (97.0, 198.5)	−3.16^b^	0.002
ALT (U/L)	26.0 (14.5, 34.0)	34.0 (19.0, 55.5)	−2.693^b^	0.007
AST (U/L)	23.0 (17.5, 31.0)	34.0 (19.5, 52.0)	−2.901^b^	0.004
γ-GT (U/L)	27.0 (16.5, 35.0)	34.0 (20.0, 51.0)	−2.098^b^	0.036
Total protein (g/L)	63.8 ± 4.4	50.0 ± 13.1	7.528^a^	<0.001
Total bilirubin (μmol/L)	10.8 (8.9, 14.2)	20.2 (13.5, 34.9)	−5.733^b^	<0.001
Hepatic fibrosis				
Hyaluronic acid (ng/mL)	50.0 (50.0, 68.4)	58.9 (50.0, 85.9)	−2.442 ^b^	0.015
Laminin (ng/mL)	30.5 (24.9, 42.3)	35.5 (22.1, 50.6)	−0.955 ^b^	0.340
Procollagen III (ng/mL)	7.4 (5.7, 8.5)	8.1 (6.6, 12.3)	−2.190 ^b^	0.028
Collagen IV (ng/mL)	48.5 (36.8, 65.4)	70.1 (58.0, 91.2)	−4.571 ^b^	<0.001

APO, adverse pregnancy outcome; UP, uneventful pregnancy; ALT, alanine aminotransferase, AST, aspartate aminotransferase; γ-GT, γ-glutamyl transpeptidase; WBC, white blood cell, RBC, red blood cell.

a *t* value.

b *Z* value.

### 3.3 LASSO regression and ROC analysis

**Figure 2:**
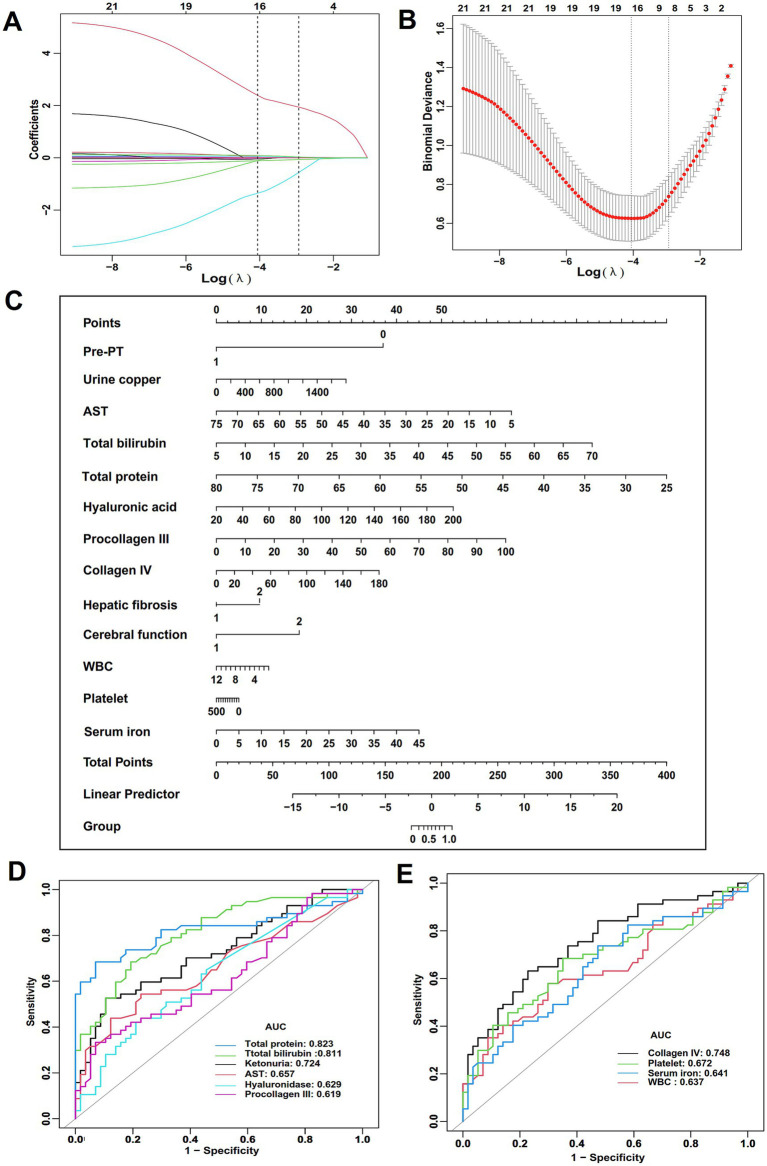
Feature selection and predictive analysis. **(A)** LASSO regression selected nonzero coefficient parameters. **(B)** LASSO regression profiles of 11 parameters. **(C)** Nomogram for predicting the probability of APOs. **(D,E)** The predictive value of a single parameters.

A LASSO regression analysis was performed on the aforementioned 16 significant variables. The final model left 11 variables, including hepatic fibrosis, cerebral function, pre-pregnancy copper-chelating therapy, WBC, platelet, total bilirubin, total protein, AST, hyaluronic acid, procollagen III, collagen IV, 24-h urinary copper, and serum iron (Figures 2A,B). These results were also visualized using a nomogram (Figure 2C). Among these variables, total protein demonstrated the highest predictive value (AUC = 0.823, Table 3; Figure 2D), while WBC showed the lowest value (AUC = 0.637, Table 3; Figure 2E).

**Table 3:** Receiver operating characteristic analysis.

Characteristics	Best threshold	AUC (95% CI)	Specificity	Sensitivity	Accuracy	Positive likelihood ratio	Negative likelihood ratio	Positive predictive value	Negative predictive value
Urine copper	738.0	0.724 (0.631, 0.817)	0.895	0.526	0.711	5.000	0.529	0.833	0.654
Serum iron	10.5	0.641 (0.539, 0.743)	0.526	0.737	0.632	1.556	0.500	0.609	0.667
AST	31.5	0.658 (0.556, 0.759)	0.772	0.544	0.658	2.385	0.591	0.705	0.629
Total bilirubin	15.0	0.811 (0.733, 0.890)	0.807	0.684	0.746	3.546	0.391	0.780	0.719
Total protein	58.0	0.823 (0.740, 0.906)	0.930	0.684	0.807	9.750	0.340	0.907	0.747
Hyaluronic acid	68.6	0.629 (0.530, 0.729)	0.772	0.439	0.605	1.923	0.727	0.658	0.579
Procollagen III	11.2	0.619 (0.515, 0.723)	0.930	0.333	0.632	4.750	0.717	0.826	0.582
Collagen IV	65.6	0.748 (0.659, 0.838)	0.772	0.632	0.702	2.769	0.477	0.735	0.677
WBC	4.2	0.637 (0.534, 0.740)	0.702	0.579	0.640	1.941	0.600	0.660	0.625
Platelet	152.5	0.672 (0.571, 0.772)	0.649	0.684	0.667	1.950	0.487	0.661	0.673
Combined	NA	0.979 (0.957, 1.000)	0.965	0.930	0.947	26.500	0.073	0.934	0.932

AUC, area under the curve; AST, aspartate aminotransferase; CI, confidence interval; WBC, white blood cell.

### 3.4 ML performance in differentiating between APO and UP

**Figure 3:**
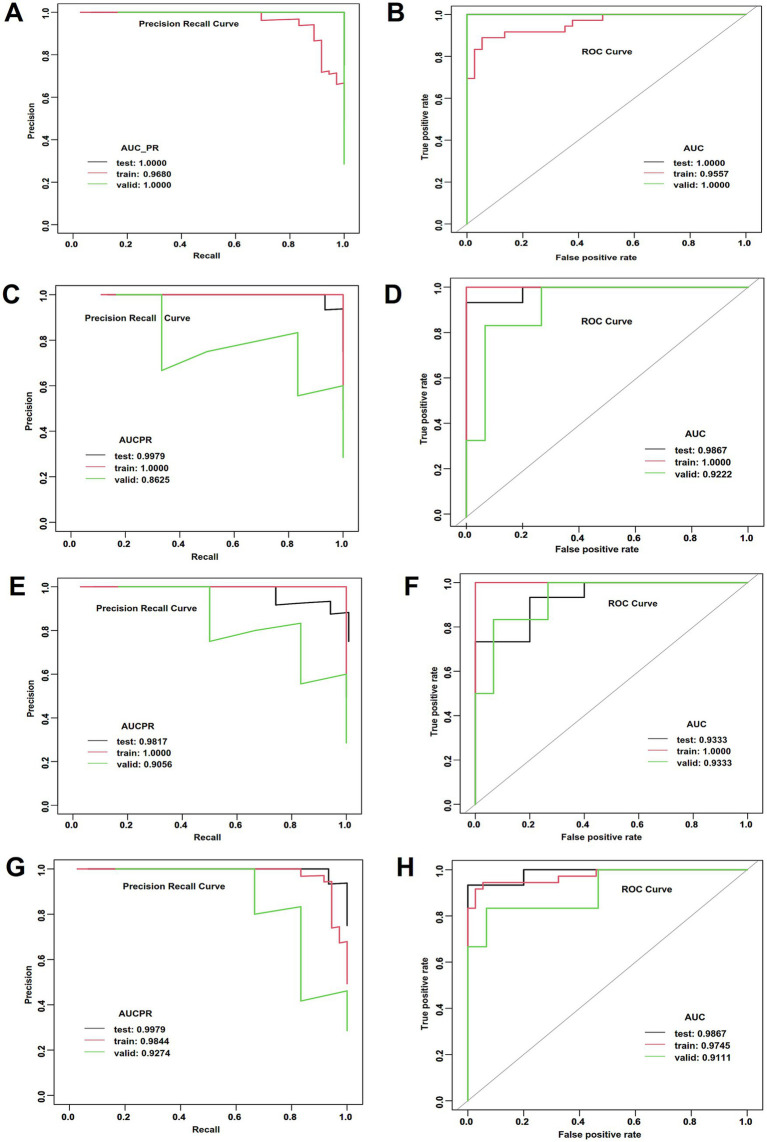
ML performance in the estimation of APOs. **(A)** AUCPR of the deep learning model. **(B)** AUCs of the deep learning model. **(C)** AUCPR of the RF model. **(D)** AUCs of the RF model. **(E)** AUCPR of the GBM model. **(F)** AUCs of the GBM model. **(G)** AUCPR of the deep learning GLM model. **(H)** AUCs of the GLM model.

The performance of the four ML models is summarized in Tables 4, 5 and Figures 3A–H. The GLM achieved the highest accuracy (0.850) and demonstrated optimal model performance in AUCPR (0.998) and AUC (0.987) (Table 4; Figures 3G,H), suggesting that its predictions are the most reliable and accurate. Minimal differences were observed for all metrics (e.g., accuracy, AUC, and sensitivity) between the testing set, training set, and validation set of the GLM model (Table 4), which reflects extremely high stability and an absence of overfitting (Table 5). The RF exhibited an accuracy (0.800) comparable to that of DL and also had the highest AUCPR (0.998) and AUC (0.987) (Table 4; Figures 3C,D). The GBM had perfect accuracy (0.986), AUC (1.000) in the training set (Tables 4, 5; Figures 3E,F). but declined in the testing set (accuracy: 0.750, Tables 4, 5), suggesting that the model over-memorized the training data, leading to poor performance on new data. The DL model showed a high AUC (1.000) in the testing set but a negative R square (Tables 4, 5; Figures 3A,B), suggesting that poor generalization ability and unreliable results of the DL model. The performance evaluations of ML models are shown in Table 5 and Supplementary Table 1.

**Table 4:** The performance of the four machine learning models.

Characteristics	Accuracy			AUC			AUCPR			Sensitivity			Specificity			Positive predictive value			Negative predictive value		
	Test	Train	Valid	Test	Train	Valid	Test	Train	Valid	Test	Train	Valid	Test	Train	Valid	Test	Train	Valid	Test	Train	Valid
DL	0.800	0.877	1.000	1.000	0.956	1.000	1.000	0.968	1.000	0.733	0.778	1.000	1.000	0.973	1.000	1.000	0.966	1.000	0.556	0.818	1.000
RF	0.800	0.932	0.905	0.987	1.000	0.922	0.998	1.000	0.863	0.733	0.861	0.833	1.000	1.000	0.933	1.000	1.000	0.833	0.556	0.881	0.933
GBM	0.750	0.986	0.905	0.933	1.000	0.933	0.982	1.000	0.906	0.667	0.972	0.833	1.000	1.000	0.933	1.000	1.000	0.833	0.500	0.974	0.933
GLM	0.850	0.890	0.905	0.987	0.975	0.911	0.998	0.984	0.927	0.800	0.778	0.833	1.000	1.000	0.933	1.000	1.000	0.833	0.625	0.822	0.933

AUC, area under the curve for receiver operating characteristic; AUCPR, AUC for precision-recall; DL, deep learning; GBM, gradient boosting machine; GLM, generalized linear model; RF, random forest.

**Table 5:** Evaluation of machine learning models performance.

Characteristics	RMSE			MSE			R square		
	Test	Train	Valid	Test	Train	Valid	Test	Train	Valid
DL	0.483	0.421	0.326	0.233	0.177	0.106	−0.244	0.291	0.479
RF	0.270	0.354	0.374	0.073	0.126	0.140	0.611	0.498	0.315
GBM	0.077	0.314	0.395	0.099	0.006	0.156	0.474	0.976	0.235
GLM	0.231	0.245	0.342	0.053	0.060	0.117	0.715	0.760	0.428

DL, deep learning; GBM, gradient boosting machine; GLM, generalized linear model; RF, random forest; MSE, mean square error; RMSE, root mean square error.

### 3.5 Stratified analysis for cerebral function

**Figure 4:**
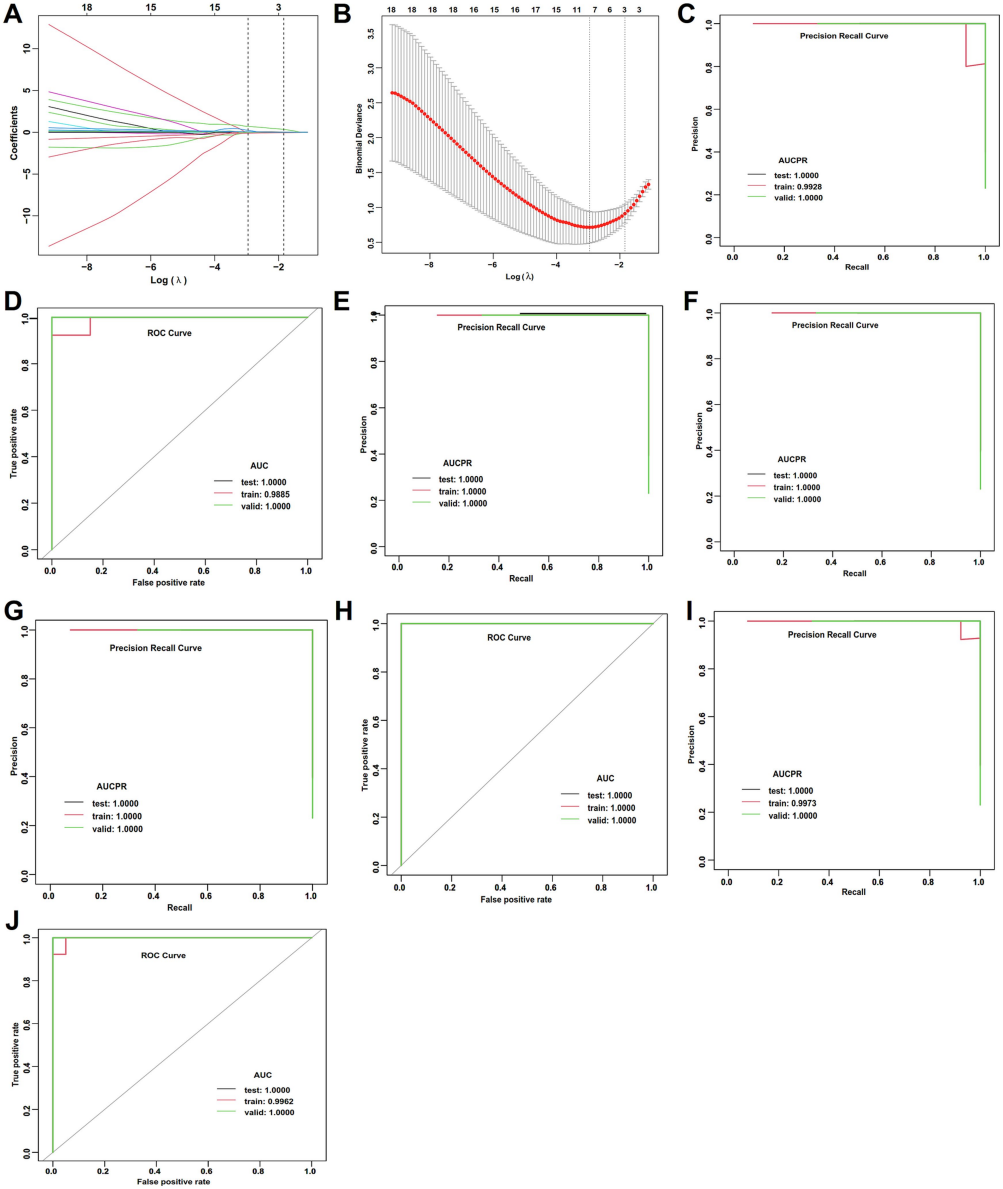
LASSO regression and ML performance in the estimation of APOs of normal cerebral function of WD. **(A)** LASSO regression selected nonzero coefficient parameters. **(B)** LASSO regression profiles. **(C)** AUCPR of the deep learning model. **(D)** AUCs of the deep learning model. **(E)** AUCPR of the RF model. **(F)** AUCs of the RF model. **(G)** AUCPR of the GBM model. **(H)** AUCs of the GBM model. **(I)** AUCPR of the deep learning GLM model. **(J)** AUCs of the GLM model.

Of the 114 patients with WD, 51 had normal cerebral function and 63 had abnormal cerebral function. Based on the findings of univariate analysis (Supplementary Tables 2, 3), LASSO regression and ROC analysis identified disease duration, pre-pregnancy treatment, total bilirubin, total protein, collagen IV, Kayser-Fleischer rings, and serum iron as independent risk factors in WD patients with normal cerebral function (Figures 4A,B). The GLM achieved the best performance on the testing set, including accuracy (1.000), AUC (1.000), and sensitivity (1.000) (Figures 4I,J; Supplementary Table 4), indicating highly reliable classification capability. All metrics demonstrated high consistency across the training, validation, and testing sets, with no significant overfitting (Supplementary Tables 5, 6).

The DL model ranked second. It achieved perfect classification metrics (accuracy, AUC, and sensitivity all 1.000) in the testing set (Supplementary Table 4; Figures 4C,D), indicating strong classification capability, along with high accuracy in regression prediction (Supplementary Tables 4–6). However, some metrics (e.g., accuracy 0.939) were slightly lower in the training set than those in the testing set (Supplementary Tables 4, 5). The RF model had substantially lower accuracy (0.800) and sensitivity (0.500) on the testing set compared to other models (Supplementary Table 4; Figures 4E,F), indicating limited classification ability for new samples. The GBM showed significantly higher regression error in the testing set (Supplementary Tables 5, 6; Figures 4G,H), suggesting overfitting.

**Figure 5:**
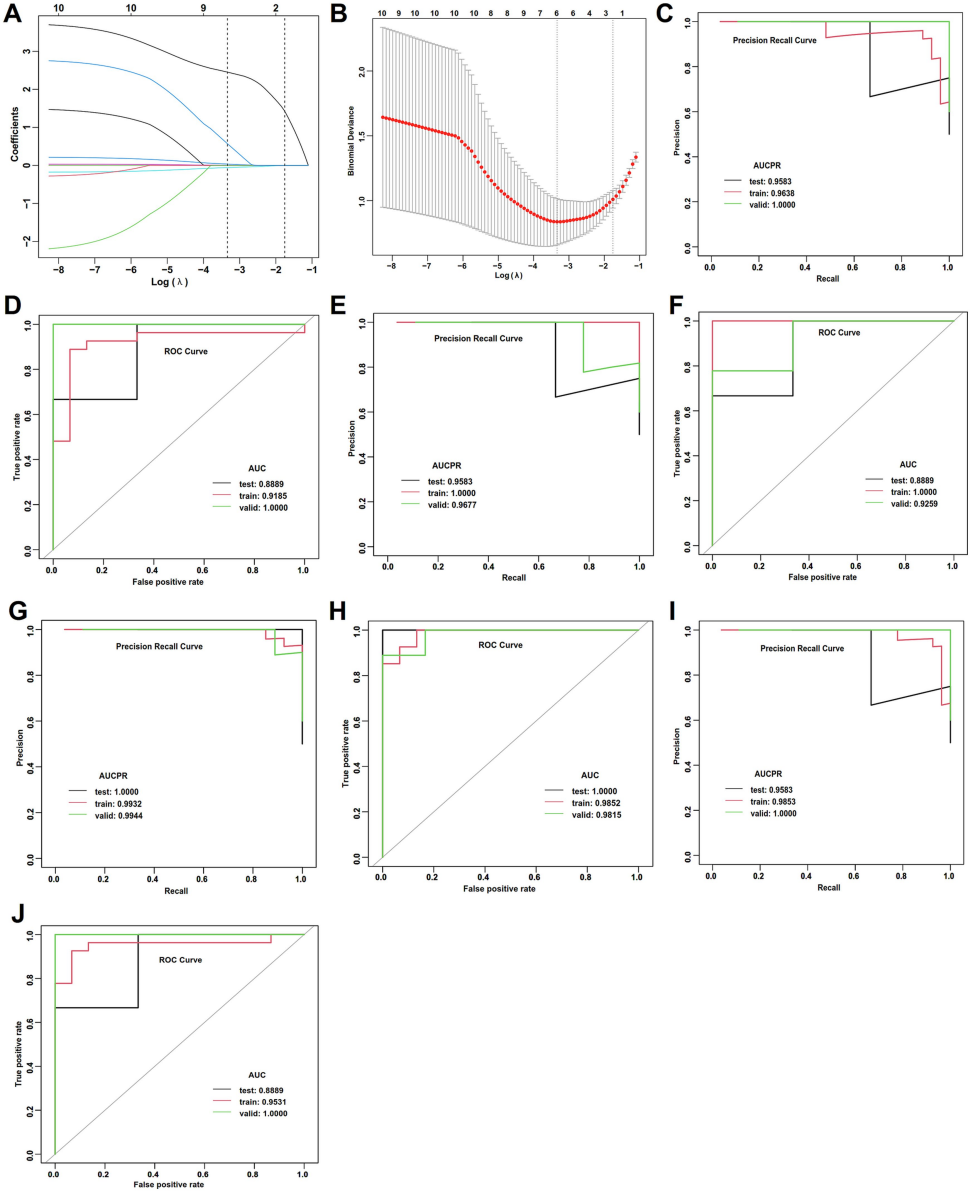
LASSO regression and ML performance in the estimation of APOs of abnormal cerebral function of WD. **(A)** LASSO regression selected nonzero coefficient parameters. **(B)** LASSO regression profiles. **(C)** AUCPR of the deep learning model. **(D)** AUCs of the deep learning model. **(E)** AUCPR of the RF model. **(F)** AUCs of the RF model. **(G)** AUCPR of the GBM model. **(H)** AUCs of the GBM model. **(I)** AUCPR of the deep learning GLM model. **(J)** AUCs of the GLM model.

In WD patients with abnormal cerebral function, LASSO regression and ROC analysis were conducted on variables with significant differences in the univariate analysis (Supplementary Tables 2, 3; Figures 5A,B). The GBM model achieved the highest accuracy (1.000), AUC (1.000), and sensitivity (1.000) in the testing set (Supplementary Tables 4–6; Figures 5G,H), indicating its superior and stable performance. The GLM model demonstrated favorable accuracy (0.833), AUC (0.889), and AUCPR (0.958) in the testing set (Supplementary Tables 4–6; Figures 5I,J), with stable regression metrics. Whereas DL and RF models showed significantly lower performance on key metrics such as accuracy (0.643) and sensitivity (0.482) in the training set compared to the testing and validation sets (Supplementary Tables 4–6; Figures 5C–F), suggesting the presence of overfitting in these models.

### 3.6 Stratified analysis for hepatic fibrosis

**Figure 6:**
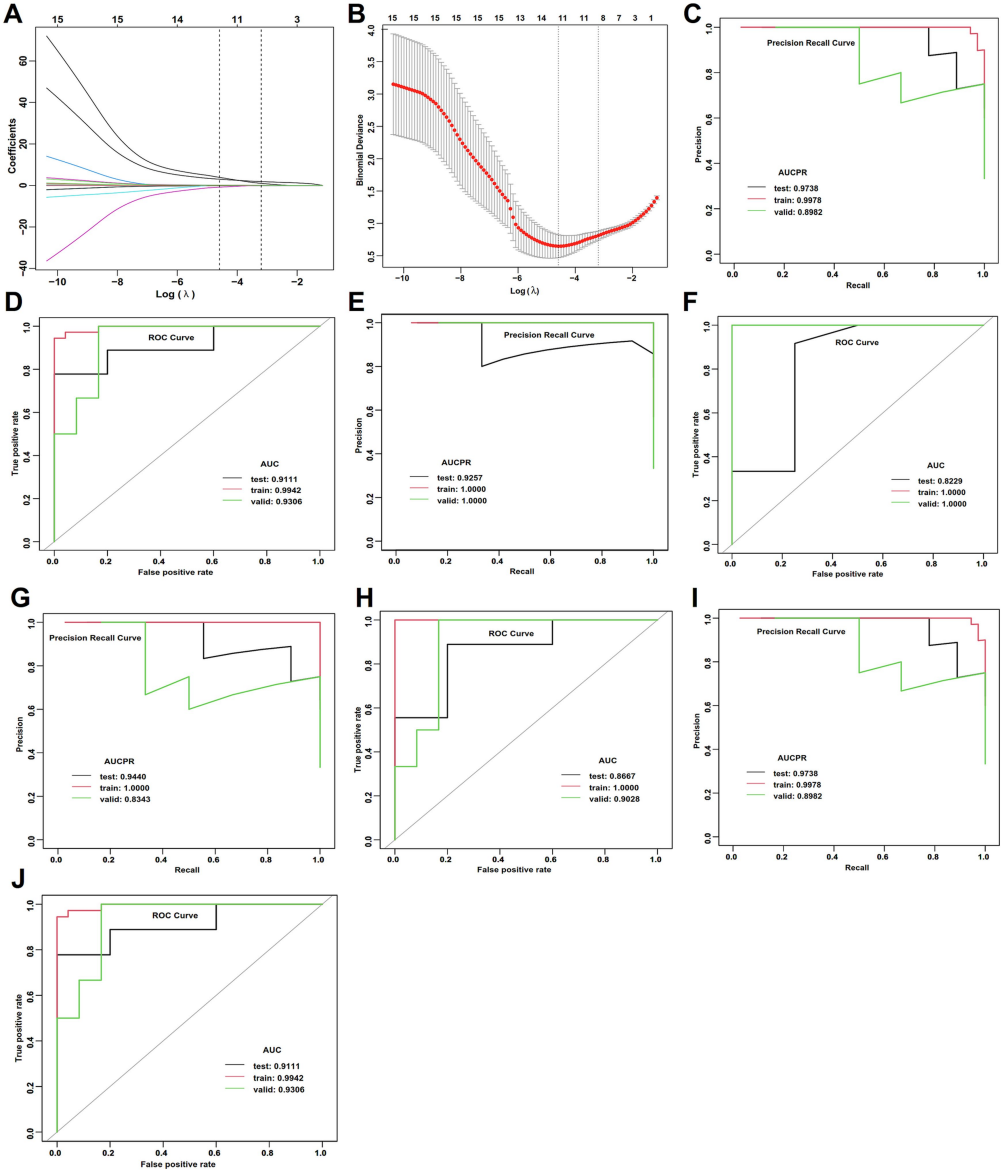
LASSO regression and ML performance in the estimation of APOs of in WD patients without hepatic fibrosis. **(A)** LASSO regression selected nonzero coefficient parameters. **(B)** LASSO regression profiles. **(C)** AUCPR of the deep learning model. **(D)** AUCs of the deep learning model. **(E)** AUCPR of the RF model. **(F)** AUCs of the RF model. **(G)** AUCPR of the GBM model. **(H)** AUCs of the GBM model. **(I)** AUCPR of the deep learning GLM model. **(J)** AUCs of the GLM model.

Of 114 patients with WD, 92 cases with and 22 without hepatic fibrosis. For WD patients with hepatic fibrosis, LASSO regression and ROC analysis were conducted on the variables with significant differences in the univariate analysis (Supplementary Tables 2, 3; Figures 6A,B). Both DL and GLM achieved the best accuracy (0.857), AUC (0.911), and AUCPR (0.974), with all metrics demonstrating high consistency across the training, testing, and validation sets (Supplementary Tables 4–6; Figures 6I–J). These findings suggested that the two models have superior, reliable and stable performance on new data. The GBM had moderate accuracy (0.857) and AUCPR (0.944) in the testing set but exhibited overfitting (Supplementary Tables 4–6; Figures 6A,B). The RF achieved perfect scores for all metrics in the training set (accuracy: 1.000, AUC: 1.000), but these metrics declined significantly in the testing set (accuracy: 0.625, AUC: 0.823), accompanied by a negative R square value (Supplementary Tables 4–6), indicating severe overfitting and poor generalization capability.

**Figure 7:**
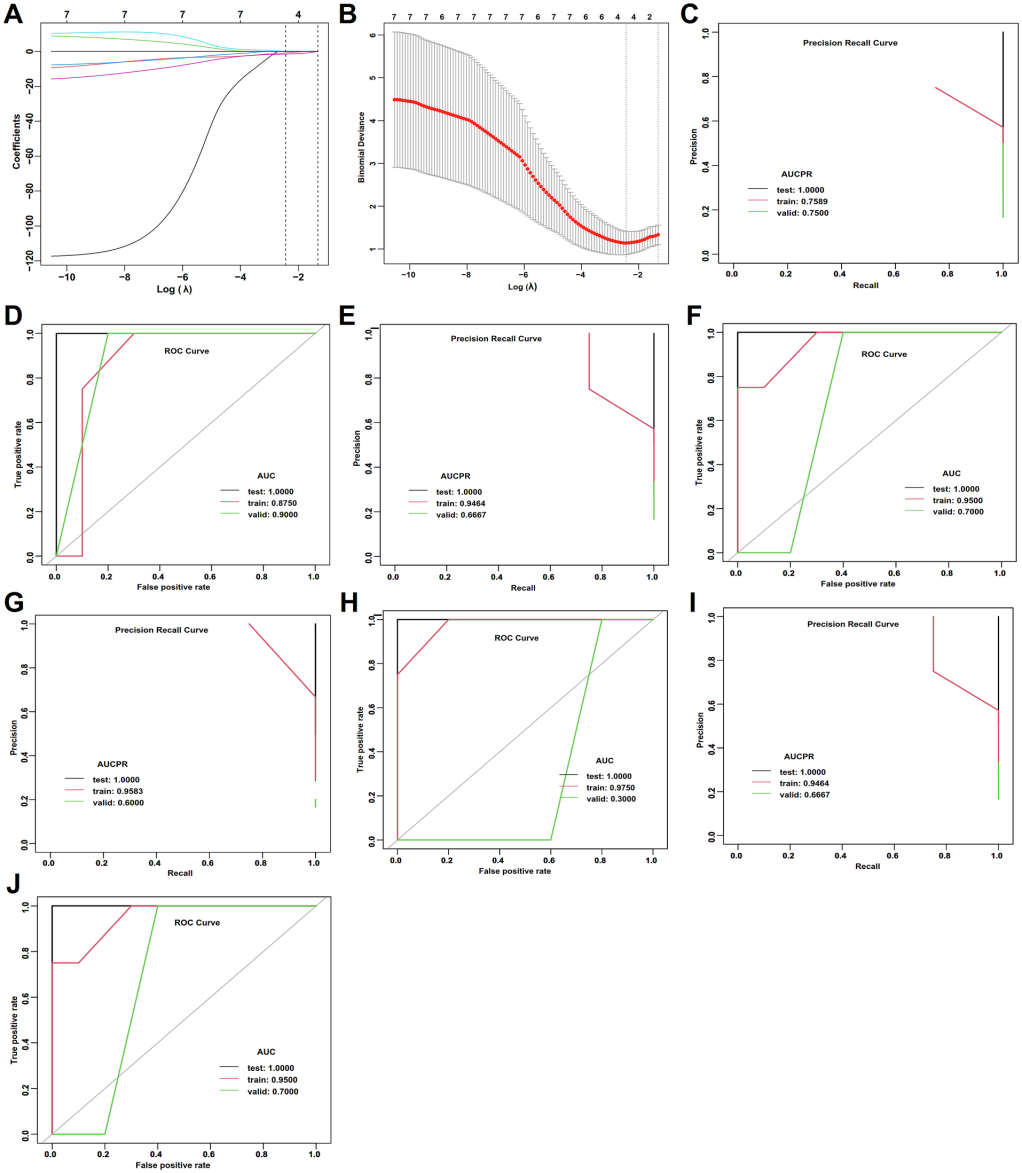
LASSO regression and ML performance in the estimation of APOs of in WD patients with hepatic fibrosis. **(A)** LASSO regression selected nonzero coefficient parameters. **(B)** LASSO regression profiles. **(C)** AUCPR of the deep learning model. **(D)** AUCs of the deep learning model. **(E)** AUCPR of the RF model. **(F)** AUCs of the RF model. **(G)** AUCPR of the GBM model. **(H)** AUCs of the GBM model. **(I)** AUCPR of the deep learning GLM model. **(J)** AUCs of the GLM model.

Although this study only enrolled 22 WD patients without hepatic fibrosis, we proceeded to develop ML models. Cerebral function and Kayser-Fleischer rings demonstrated statistical significance in the univariate analysis, followed by LASSO regression, ROC analysis, and ML models (Supplementary Tables 1–6; Figures 7A–J). However, all models performed bad performance in the testing set and exhibited significant overfitting. In addition, sensitivity analysis based on 5-fold cross-validation identified the GLM as the optimal model, achieving the best classification performance and predictive calibration, with the lowest risk of overfitting. In contrast, RF and GBM demonstrated significant overfitting (Supplementary Table 7).

## 4 Discussion

In the most patients with WD, diagnosis is established before or during their reproductive age (20). It has raised reasonable concerns regarding fertility issues, maternal disease exacerbation, disease duration, teratogenicity of chelating agents, and pregnancy outcomes (4, 8, 20). Therefore, it is important to develop novel tools for identifying APOs in patients with WD. In this study, we comprehensively investigated the microelements and biochemical markers for APOs in patients with WD and evaluated the performance of various ML models in predicting APOs. Our results suggested that disease duration, inadequate pre-pregnancy decoppering therapy, abnormal copper metabolism (especially 24 h-urine copper and serum iron), and elevated liver fibrosis biomarkers (i.e., hyaluronic acid, procollagen III, and collagen IV) (15) are significantly associated with an increased risk of APOs. Among the four ML models, the GLM exhibited the best predictive accuracy and stability, whereas the RF and the GBM models had significant overfitting.

This study enrolled 114 patients with WD, comprising 57 with APO and 57 with UP. LASSO regression was used for feature selection and to mitigate potential multicollinearity between independent variables. Among the selected 11 key variables, serum iron, hepatic fibrosis, cerebral WD, total bilirubin, and collagen IV were identified as independent risk factors. These APOs-related factors identified in this study are highly consistent with the risk of WD (12, 21, 22). The identification of cerebral function and hepatic fibrosis as the major risk factors reflect the multisystem involvement characteristic of WD (8). Cerebral WD is often associated with increased copper accumulation and neurological dysfunction, which may increase the risk of APOs (23). Liver fibrosis indicates a significant impairment of liver function, potentially leading to insufficient protein synthesis and decreased detoxification ability, thereby affecting fetal development (24). Abnormal copper metabolism is implicated in the occurrence of APOs (15, 21). First, an increase in 24-h urinary copper levels in APOs suggests systemic copper overload, which is associated with the accumulation of tissue copper (11, 25). Second, elevated serum iron levels may exacerbate oxidative damage by promoting free radical generation, consistent with previous studies on the impact of metal ion metabolism on APOs (26, 27). Additionally, increased levels of liver fibrosis markers, such as pro-collagen III and collagen IV, further support the role of chronic liver injury in APOs. These biomarkers are key indicators during the progression of liver fibrosis (28). Third, association between inadequate decoppering pre-pregnancy therapy and APOs. Pre-pregnancy decoppering therapy can reduce tissue copper load and alleviate the damage caused by copper toxicity to both the mother and fetus (20). Our findings suggested that standardized pre-pregnancy treatment is of great significance for female patients with WD, especially those planning pregnancy.

In recent years, various novel clinical diagnostic and predictive tools for chronic diseases have been developed using sophisticated ML models (16–18). In this study, four machine learning models (i.e., GLM, RF, GBM, and DL) were firstly used for predicting the risk of APOs. The GLM showed the best performances in the testing set, with a low error value indicating robust generalization capability and a low risk of overfitting (16). In the GLM, elevated 24-h urinary copper and serum iron levels were independently associated with APOs. Clinically, elevated urinary copper is a direct biomarker of systemic copper overload in patients with WD, while elevated serum iron may indicate homeostatic dysregulation of systemic iron. In addition, the hepatic fibrosis biomarker type IV collagen was left in our model, which may indicate an advanced systemic disease state characterized by impaired hepatic synthetic function and altered metabolic capacity, thereby creating an unfavorable maternal environment for sustaining pregnancy. Therefore, our model identifies a predictive signature centered on dysregulated metal ion homeostasis and hepatic injury, providing a quantitative basis for the long-standing clinical observation that poorly controlled WD increases pregnancy risk. Although the remaining three ML models performed well in the training set, they exhibited significant overfitting. It may be due to these models over-learned the noise in the training data rather than the underlying biological patterns. These findings underscore the importance of balancing model complexity and data characteristics when developing clinical prediction models (29). This may be attributed to these models excessively learning noise from the training data rather than potential biological patterns.

Findings of the stratified analysis further revealed the effect of WD heterogeneity on ML performance. In the abnormal cerebral function subgroup, GBM achieved the best performance, whereas GLM had the highest predictive accuracy in the normal cerebral function subgroup. This discrepancy may be attributed to the varying weights of risk factors across different subgroups (16). For example, in patients with cerebral dysfunction, Kayser–Fleischer rings and serum copper levels showed higher predictive value for APOs, whereas hepatic fibrosis-related biomarkers exhibited stronger discriminative power in the normal cerebral function subgroup (30). In the hepatic fibrosis subgroup, both DL and GLM performed excellent performance, likely because hepatic fibrosis-related biomarkers provided rich predictive information. However, in the non-hepatic fibrosis subgroup (only 22 cases), all models exhibited poor performance and overfitting, highlighting the challenge of extremely small-sample sizes for model stability. These findings are consistent with the requirements for data scale and representativeness in conducting ML (16–18, 29), suggesting the need for future multi-center collaborations to expand the sample sizes. Additionally, our analysis revealed that the key variables included in the four ML models differed (16). These differences suggest that different microelements and biochemical markers have unique discriminative capabilities for specific disease subtypes (16).

This study has several strengths that enhance the credibility and clinical relevance of the findings. First, this investigation was conducted on a well-characterized cohort of patients with WD, providing robust power for analyzing pregnancy outcomes through the integration of detailed microelements, clinical phenotypes, and hepatic fibrosis biomarkers. Second, multiple ML algorithms were performed, combined with rigorous validation and stratified analysis. The most reliable ML model was identified by comparing evaluation results of different models, and the limitations associated with the use of a small-sample size were also evaluated (16). Finally, this study provided a more practical insight for clinical practice by demonstrating a simple yet reliable stratified analysis, laying a foundation for personalized management strategies based on cerebral function and liver fibrosis for patients with WD during pregnancy. Nevertheless, several limitations should also be considered. First, the retrospective cross-sectional design employed in this study limits the ability to definitively establish the temporal sequence between predictive factors and APOs, suggesting that these findings represent associations rather than causal relationships. Second, although LASSO regression was used for feature selection and multiple clinical factors were adjusted prior to constructing the ML models, unmeasured confounding may still exist, potentially biasing the results. Finally, as a single-center study, the sample sizes within subgroups (especially WD patients without hepatic fibrosis group) were very limited, resulting in unstable and potentially over-optimistic model performance. Therefore, these results should be interpreted with caution. Future researches involving larger sample sizes, prospective designs, and multi-center collaborations are warranted to systematically collect genetic, environmental, and detailed treatment data to validate the efficacy of the ML models.

In conclusion, microelements and biochemical markers play important roles in the occurrence of APOs in patients with WD. ML models, especially the GLM model, can effectively predict the risk of APOs based on these key biomarkers. GLM exhibited superior generalization capability and robustness, making it the most reliable predictive tool. Stratified analysis revealed that model performance is highly dependent on patient subgroups. These findings support the integration of patient stratification in clinical prediction models for patients with WD, and the GLM represents a promising tool for developing clinical decision support systems to achieve personalized risk assessment and improve pregnancy management.

## Data Availability

The raw data are available from the corresponding author upon reasonable request. Requests to access these datasets should be directed to zhangxu@ahmu.edu.cn.
