# Correction: Microelements and biochemical biomarkers-based machine learning for predicting adverse pregnancy outcomes in Wilson's disease: risk stratification by integrating hepatic fibrosis and cerebral function

**DOI:** 10.3389/fnut.2026.1813204

**Published:** 2026-03-11

**Authors:** Juan Wang, Qing-qing Ming, Yong-guang Shi, Yin Xu, Jun-cang Wu, Xu-en Yu, Xu Zhang

**Affiliations:** 1 Department of Neurology, Hefei Hospital Affiliated to Anhui Medical University (Hefei Second People's Hospital), Department of Health Management Center, First Affiliated Hospital of Anhui Medical University, Hefei, China; 2 Institute of Neurology, Anhui University of Chinese Medicine, Department of Neurology, Affiliated Hospital of the Neurology Research Institute of Anhui University of Chinese Medicine, Hefei, China

**Keywords:** adverse pregnancy outcomes, generalized linear model, machine learning, microelements, uneventful pregnancy, Wilson's disease

The affiliation “Institute of Neurology, Anhui University of Chinese Medicine, Department of Neurology, Affiliated Hospital of the Neurology Research Institute of Anhui University of Chinese Medicine, Hefei, China” was omitted for authors Qing-qing Ming, Yong-guang Shi, Yin Xu, and Xu-en Yu. This affiliation has now been added for these authors.

The affiliation “Department of Neurology, Hefei Hospital Affiliated to Anhui Medical University (Hefei Second People's Hospital), Department of Health Management Center, First Affiliated Hospital of Anhui Medical University, Hefei, China” was erroneously assigned for authors Qing-qing Ming, Yong-guang Shi, Yin Xu, and Xu-en Yu. This affiliation has now been removed for these authors.

The original version of this article has been updated.

