# Supplementary material for: Microelements and biochemical biomarkers-based machine learning for predicting adverse pregnancy outcomes in Wilson’s disease: risk stratification by integrating hepatic fibrosis and cerebral function
Source: Front Nutr. 2026 Feb 17;13:1768588. doi: 10.3389/fnut.2026.1768588 (PMC12954587; doi:10.3389/fnut.2026.1768588)
Supplement: Supplementary file 1 [file Supplementary_file_1.docx]

**Supplementary Materials**

**Description of four machine learning (ML) models**

Deep learning (DL), an artificial neural network (ANN), is an extension of the single-layer perceptron. Its most significant feature is the inclusion of multiple hidden layers in addition to the input and output layers. The first layer of an DL is termed the input layer, the intermediate layers are the hidden layers, and the final layer is the output layer. The DL does not prescribe a fixed number of hidden layers; thus, an appropriate number can be selected based on the specific processing requirements. Furthermore, there are no restrictions on the number of neurons in each hidden layer or the output layer.

Random forest (RF) is a machine learning algorithm utilized for classification, regression, and feature selection. It is a synthesis learning method that achieves more accurate predictions by combining multiple decision trees. In a RF model, each decision tree is trained on a randomly selected subset of data and a randomly selected subset of features, which reduces overfitting and enhance model accuracy. During prediction, each tree provides a classification (i.e., the tree "votes" for a class), and the final forest selection is determined by the class receiving the most votes.

Generalized Linear Model (GLM) is an extension of the linear model, employed to investigate the relationship between dependent and independent variables. Compared to logistic regression, GLM can handle independent variables that do not conform to the assumption of a normal distribution and can be applied to both categorical and continuous variables. A GLM comprises three components including the random component, the systematic component, and the link function.

Gradient Boosting Machine (GBM) is a boosting algorithm composed of multiple learners. The core concept of gradient boosting is to sequentially generate multiple weak learners. The objective of each weak learner is to fit the loss function of the previously accumulated model, thereby reducing the loss of the cumulative model after the weak learner is added. This process is repeated for multiple iterations until the error is minimized or the maximum number of iterations is reached. Gradient boosting involves three elements, i.e., optimization of the loss function, prediction by the weak learners, and the addition of weak learners to the model to minimize the loss function.

## Model training

Ultimately, 114 patients with WD were included and 24 features were recorded and measured (Table 1-2 & Supplementary Table 2). Of the 24 variables, seven were characteristics of patients with WD (i.e., age at conception, disease duration, disease subtype, pre-pregnancy treatment, Kayser-Fleischer, hepatic fibrosis, and cerebral function), five microelement-associated biomarkers (i.e., serum copper, ceruloplasmin, urine copper, serum zinc, and serum iron), eight Biochemical markers (WBC, RBC, platelet, ALT, AST, γ-GT, total protein, and total bilirubin), four hepatic fibrosis-associated biomarkers (hyaluronic acid, laminin, procollagen III, and collagen IV). Subsequently, a random 75% of dataset was used for ML training models, 15% for testing the models, and the remaining 15% for validation set. Even though the model being divided into 75% training set, 15% testing set and 5% validation set, dataset is still balanced and there is no missing value. Finally, we fit DL, RF, GBM, and GLM to the training sets, and each model was used to generate predictions for the testing and validation data. Meanwhile, subgroups of cerebral function (normal cerebral function *vs.* abnormal cerebral dysfunction) and hepatic fibrosis (with *vs.* without hepatic fibrosis) were also used to generate predictions.

**Supplementary Table 1. Performance evaluation of machine learning**

|  | Brier Score  (95% CI) | Calibration-in-the-large  (95% CI) | Calibration Slope  (95% CI) |
| --- | --- | --- | --- |
| **Overall** |  |  |  |
| DL | 0.141 (0.118, 0.164) | 0.031 (-0.012, 0.074) | 1.081 (0.924, 1.238) |
| RF | 0.159 (0.136, 0.182) | 0.039 (-0.005, 0.083) | 1.025 (0.877, 1.173) |
| GBM | 0.151 (0.127, 0.175) | 0.017 (-0.026, 0.060) | 1.126 (0.960, 1.292) |
| GLM | 0.155 (0.132, 0.178) | 0.024 (-0.019, 0.067) | 1.057 (0.909, 1.205) |

CI, confidence interval; DL, deep learning; GBM, gradient boosting machine; GLM, generalized linear model; RF, random forest.

**Supplementary Table 2. Univariate analyses of subgroups**

|  | Beta | *P* | OR (95% CI) |
| --- | --- | --- | --- |
| **Abnormal** **cerebral function** | |  |  |
| Age at conception | 0.051 | 0.455 | 1.052 (0.921, 1.201) |
| Disease duration | -0.040 | 0.300 | 0.961 (0.891, 1.036) |
| Pre_PT | -4.200 | <0.001 | 0.015 (0.002, 0.126) |
| Kayser-Fleischer | 0.660 | 0.005 | 1.935 (1.226, 3.055) |
| Hepatic fibrosis | 0.356 | 0.298 | 1.428 (0.73, 2.795) |
| Serum copper | -0.081 | 0.380 | 0.922 (0.77, 1.105) |
| Ceruloplasmin | 0.001 | 0.926 | 1.001 (0.985, 1.017) |
| Urine copper | 0.004 | 0.003 | 1.004 (1.001, 1.007) |
| Serum zinc | 0.036 | 0.313 | 1.036 (0.967, 1.111) |
| Serum iron | 0.102 | 0.059 | 1.107 (0.996, 1.231) |
| WBC | -0.222 | 0.208 | 0.801 (0.566, 1.132) |
| RBC | 0.602 | 0.378 | 1.826 (0.479, 6.961) |
| Platelet | -0.006 | 0.153 | 0.994 (0.987, 1.002) |
| ALT | 0.029 | 0.080 | 1.03 (0.997, 1.064) |
| AST | 0.039 | 0.074 | 1.04 (0.996, 1.085) |
| Total bilirubin | 0.142 | 0.005 | 1.153 (1.044, 1.273) |
| Total protein | -0.149 | 0.002 | 0.861 (0.784, 0.947) |
| Hyaluronic acid | 0.005 | 0.643 | 1.006 (0.982, 1.029) |
| Laminin | 0.004 | 0.798 | 1.004 (0.974, 1.035) |
| Procollagen III | 0.170 | 0.103 | 1.185 (0.966, 1.453) |
| Collagen IV | 0.045 | 0.002 | 1.046 (1.017, 1.076) |
| **Normal cerebral function** | |  |  |
| Age at conception | 0.037 | 0.636 | 1.037 (0.891, 1.207) |
| Disease duration | -0.115 | 0.030 | 0.892 (0.804, 0.989) |
| Pre_PT | -3.258 | <0.001 | 0.038 (0.008, 0.185) |
| Kayser-Fleischer | 0.716 | 0.004 | 2.046 (1.261, 3.319) |
| Hepatic fibrosis | 1.578 | 0.006 | 4.848 (1.578, 14.892) |
| Serum copper | 0.204 | 0.107 | 1.226 (0.957, 1.57) |
| Ceruloplasmin | 0.008 | 0.290 | 1.008 (0.993, 1.022) |
| Urine copper | 0.006 | 0.004 | 1.006 (1.002, 1.01) |
| Serum zinc | 0.009 | 0.867 | 1.009 (0.909, 1.12) |
| Serum iron | 0.112 | 0.028 | 1.119 (1.012, 1.237) |
| WBC | -0.267 | 0.213 | 0.766 (0.503, 1.165) |
| RBC | -0.982 | 0.113 | 0.374 (0.111, 1.26) |
| Platelet | -0.007 | 0.125 | 0.993 (0.985, 1.002) |
| ALT | 0.057 | 0.003 | 1.059 (1.019, 1.1) |
| AST | 0.077 | 0.001 | 1.08 (1.032, 1.131) |
| Total bilirubin | 0.154 | <0.001 | 1.167 (1.07, 1.271) |
| Total protein | -0.219 | 0.001 | 0.804 (0.71, 0.909) |
| Hyaluronic acid | 0.059 | 0.002 | 1.061 (1.023, 1.101) |
| Laminin | 0.024 | 0.117 | 1.025 (0.994, 1.056) |
| Procollagen III | 0.158 | 0.021 | 1.171 (1.024, 1.34) |
| Collagen IV | 0.081 | 0.001 | 1.084 (1.034, 1.136) |
| **With hepatic fibrosis** | |  |  |
| Age at conception | 0.087 | 0.121 | 1.091 (0.977, 1.219) |
| Disease duration | -0.049 | 0.133 | 0.952 (0.893, 1.015) |
| Pre_PT | -3.414 | <0.001 | 0.033 (0.009, 0.123) |
| Kayser-Fleischer | 0.626 | 0.001 | 1.87 (1.308, 2.672) |
| Cerebral dysfunction | 0.644 | 0.135 | 1.905 (0.818, 4.434) |
| Serum copper | 0.005 | 0.946 | 1.005 (0.871, 1.159) |
| Ceruloplasmin | 0.019 | 0.032 | 1.02 (1.002, 1.038) |
| Urine copper | 0.004 | <0.001 | 1.004 (1.002, 1.007) |
| Serum zinc | 0.062 | 0.052 | 1.064 (0.999, 1.133) |
| Serum iron | 0.087 | 0.022 | 1.091 (1.013, 1.175) |
| WBC | -0.236 | 0.087 | 0.79 (0.603, 1.034) |
| RBC | -0.036 | 0.937 | 0.965 (0.401, 2.324) |
| Platelet | -0.008 | 0.010 | 0.992 (0.986, 0.998) |
| ALT | 0.038 | 0.005 | 1.039 (1.012, 1.067) |
| AST | 0.048 | 0.003 | 1.05 (1.017, 1.084) |
| Total bilirubin | 0.169 | <0.001 | 1.184 (1.087, 1.29) |
| Total protein | -0.147 | <0.001 | 0.864 (0.806, 0.926) |
| Hyaluronic acid | 0.019 | 0.063 | 1.019 (0.999, 1.04) |
| Laminin | 0.021 | 0.110 | 1.021 (0.995, 1.047) |
| Procollagen III | 0.161 | 0.025 | 1.175 (1.021, 1.352) |
| Collagen IV | 0.052 | <0.001 | 1.053 (1.027, 1.079) |
| **Without hepatic fibrosis** | |  |  |
| Age at conception | -0.058 | 0.635 | 0.944 (0.743, 1.199) |
| Disease duration | -0.142 | 0.103 | 0.868 (0.732, 1.029) |
| Kayser-Fleischer | 1.082 | 0.016 | 2.951 (1.224, 7.113) |
| Cerebral dysfunction | 3.076 | 0.015 | 21.667 (1.802, 10.574) |
| Serum copper | 0.142 | 0.403 | 1.153 (0.826, 1.608) |
| Ceruloplasmin | -0.010 | 0.407 | 0.990 (0.966, 1.014) |
| Urine copper | 0.004 | 0.160 | 1.004 (0.998, 1.01) |
| Serum iron | 0.065 | 0.587 | 1.067 (0.844, 1.35) |
| Serum zinc | -0.205 | 0.079 | 0.815 (0.648, 1.024) |
| WBC | -0.418 | 0.382 | 0.658 (0.258, 1.68) |
| RBC | -0.359 | 0.773 | 0.699 (0.061, 7.998) |
| Platelet | 0.002 | 0.760 | 1.002 (0.991, 1.012) |
| ALT | 0.011 | 0.627 | 1.011 (0.967, 1.057) |
| AST | 0.037 | 0.223 | 1.038 (0.977, 1.103) |
| Total bilirubin | 0.064 | 0.233 | 1.066 (0.96, 1.183) |
| Total protein | -0.403 | 0.068 | 0.668 (0.434, 1.031) |
| Hyaluronic acid | 0.006 | 0.858 | 1.006 (0.944, 1.071) |
| Laminin | -0.009 | 0.731 | 0.991 (0.942, 1.043) |
| Procollagen III | 0.150 | 0.415 | 1.161 (0.81, 1.664) |
| Collagen IV | 0.011 | 0.591 | 1.011 (0.971, 1.052) |

ALT, alanine aminotransferase; AST, aspartate aminotransferase; CI, confidence interval; WBC, white blood cell, RBC, red blood cell; Pre_PT, pre-pregnancy treatment; OR, odd ratios.

**Supplementary Table 3. Receiver operating characteristic analysis of subgroups**

|  | Best threshold | AUC (95% CI) | Specificity | Sensitivity | Accuracy | Positive likelihood ratio | Negative likelihood ratio | Positive predictive value | Negative predictive value |
| --- | --- | --- | --- | --- | --- | --- | --- | --- | --- |
| **Abnormal cerebral function** | | | |  |  |  |  |  |  |
| Disease duration | 10.5 | 0.685 (0.512, 0.858) | 0.697 | 0.667 | 0.686 | 2.200 | 0.478 | 0.546 | 0.793 |
| Serum iron | 14.3 | 0.689 (0.526, 0.851) | 0.758 | 0.556 | 0.686 | 2.292 | 0.587 | 0.556 | 0.758 |
| Total bilirubin | 15.7 | 0.881 (0.771, 0.990) | 0.788 | 0.944 | 0.843 | 4.452 | 0.071 | 0.708 | 0.963 |
| Total protein | 53.8 | 0.889 (0.761,1.000) | 1.000 | 0.778 | 0.922 | Inf | 0.222 | 1.000 | 0.892 |
| Procollagen III | 11.7 | 0.629 (0.440, 0.817) | 0.970 | 0.500 | 0.804 | 16.500 | 0.516 | 0.900 | 0.781 |
| Collagen IV | 71.5 | 0.855 (0.753, 0.957) | 0.788 | 0.778 | 0.784 | 3.667 | 0.282 | 0.667 | 0.867 |
| **Normal cerebral function** | | | |  |  |  |  |  |  |
| Urine copper | 733.1 | 0.729 (0.601, 0.856) | 0.875 | 0.513 | 0.651 | 4.103 | 0.557 | 0.870 | 0.525 |
| Total bilirubin | 14.5 | 0.780 (0.665, 0.895) | 0.833 | 0.590 | 0.683 | 3.539 | 0.492 | 0.852 | 0.556 |
| Total protein | 58.2 | 0.783 (0.669, 0.897) | 0.917 | 0.615 | 0.730 | 7.385 | 0.420 | 0.923 | 0.595 |
| Collagen IV | 65.4 | 0.758 (0.639, 0.876) | 0.917 | 0.513 | 0.667 | 6.154 | 0.532 | 0.909 | 0.537 |
| **With hepatic fibrosis** | | | |  |  |  |  |  |  |
| Kayser-Fleischer | NA | 0.875 (0.723, 1.000) | 0.833 | 0.750 | 0.773 | 3.333 | 0.222 | 0.556 | 0.923 |
| Cerebral function | NA | 0.823 (0.614, 1.000) | 0.833 | 0.813 | 0.818 | 4.444 | 0.205 | 0.625 | 0.929 |
| **Without hepatic fibrosis** | | | |  |  |  |  |  |  |
| Ceruloplasmin | 35.2 | 0.615 (0.450, 0.730) | 0.415 | 0.804 | 0.630 | 1.373 | 0.473 | 0.631 | 0.630 |
| Urine copper | 738.0 | 0.733 (0.631, 0.835) | 0.878 | 0.549 | 0.696 | 4.502 | 0.514 | 0.849 | 0.610 |
| Serum iron | 14.9 | 0.642 (0.529, 0.755) | 0.854 | 0.431 | 0.620 | 2.948 | 0.666 | 0.786 | 0.547 |
| AST | 38.5 | 0.666 (0.555, 0.777) | 0.902 | 0.451 | 0.652 | 4.623 | 0.608 | 0.852 | 0.569 |
| Total bilirubin | 15.0 | 0.835 (0.754, 0.916) | 0.854 | 0.706 | 0.772 | 4.824 | 0.345 | 0.857 | 0.700 |
| Total protein | 58.0 | 0.803 (0.706, 0.900) | 0.927 | 0.706 | 0.804 | 9.647 | 0.317 | 0.923 | 0.717 |
| Procollagen III | 11.3 | 0.642 (0.530, 0.755) | 0.951 | 0.333 | 0.609 | 6.833 | 0.701 | 0.895 | 0.534 |
| Collagen IV | 67.2 | 0.787 (0.693, 0.880) | 0.829 | 0.628 | 0.717 | 3.675 | 0.449 | 0.821 | 0.642 |

AUC, area under the curve; AST, aspartate aminotransferase; CI, confidence interval; WBC, white blood cell; NA, not available; Inf, infinity.

**Supplementary Table 4. The performance of machine learning models in** **stratified analyses**

|  | Accuracy | | | AUC | | | AUCPR | | | Sensitivity | | | Specificity | | | PPV | | | NPV | | |
| --- | --- | --- | --- | --- | --- | --- | --- | --- | --- | --- | --- | --- | --- | --- | --- | --- | --- | --- | --- | --- | --- |
|  | Test | Train | Valid | Test | Train | Valid | Test | Train | Valid | Test | Train | Valid | Test | Train | Valid | Test | Train | Valid | Test | Train | Valid |
| **Abnormal cerebral function** | | | |  |  |  |  |  |  |  |  |  |  |  |  |  |  |  |  |  |  |
| DL | 1.000 | 0.939 | 1.000 | 1.000 | 0.989 | 1.000 | 1.000 | 0.993 | 1.000 | 1.000 | 0.846 | 1.000 | 1.000 | 1.000 | 1.000 | 1.000 | 1.000 | 1.000 | 1.000 | 0.909 | 1.000 |
| RF | 0.800 | 0.970 | 1.000 | 1.000 | 1.000 | 1.000 | 1.000 | 1.000 | 1.000 | 0.500 | 0.923 | 1.000 | 1.000 | 1.000 | 1.000 | 1.000 | 1.000 | 1.000 | 0.750 | 0.952 | 1.000 |
| GBM | 1.000 | 1.000 | 1.000 | 1.000 | 1.000 | 1.000 | 1.000 | 1.000 | 1.000 | 1.000 | 1.000 | 1.000 | 1.000 | 1.000 | 1.000 | 1.000 | 1.000 | 1.000 | 1.000 | 1.000 | 1.000 |
| GLM | 1.000 | 0.939 | 1.000 | 1.000 | 0.996 | 1.000 | 1.000 | 0.997 | 1.000 | 1.000 | 1.000 | 1.000 | 1.000 | 0.900 | 1.000 | 1.000 | 0.887 | 1.000 | 1.000 | 1.000 | 1.000 |
| **Normal cerebral function** | | | |  |  |  |  |  |  |  |  |  |  |  |  |  |  |  |  |  |  |
| DL | 0.833 | 0.643 | 1.000 | 0.889 | 0.919 | 1.000 | 0.958 | 0.964 | 1.000 | 0.667 | 0.482 | 1.000 | 1.000 | 0.933 | 1.000 | 1.000 | 0.929 | 1.000 | 0.750 | 0.500 | 1.000 |
| RF | 0.667 | 1.000 | 0.867 | 0.889 | 1.000 | 0.926 | 0.958 | 1.000 | 0.968 | 0.667 | 1.000 | 1.000 | 0.667 | 1.000 | 0.667 | 0.667 | 1.000 | 0.818 | 0.667 | 1.000 | 1.000 |
| GBM | 1.000 | ,929 | 0.933 | 1.000 | 0.985 | 0.982 | 1.000 | 0.993 | 0.994 | 1.000 | 0.963 | 1.000 | 1.000 | 0.867 | 0.833 | 1.000 | 0.929 | 0.900 | 1.000 | 0.929 | 1.000 |
| GLM | 0.833 | 0.833 | 1.000 | 0.889 | 0.953 | 1.000 | 0.958 | 0.985 | 1.000 | 0.667 | 0.778 | 1.000 | 1.000 | 0.933 | 1.000 | 1.000 | 0.955 | 1.000 | 0.750 | 0.700 | 1.000 |
| **With hepatic fibrosis** | | | |  |  |  |  |  |  |  |  |  |  |  |  |  |  |  |  |  |  |
| DL | 0.500 | 0.714 | 0.833 | 1.000 | 0.875 | 0.900 | 1.000 | 0.759 | 0.750 | <0.001 | <0.001 | 1.000 | 1.000 | 1.000 | 0.800 | NA | NA | 0.500 | 0.500 | 0.714 | 1.000 |
| RF | 0.500 | 0.929 | 0.667 | 1.000 | 0.950 | 0.700 | 1.000 | 0.946 | 0.667 | 1.000 | 0.750 | 1.000 | 1.000 | 1.000 | 0.600 | NA | 1.000 | 0.333 | 0.500 | 0.909 | 1.000 |
| GBM | 0.500 | 0.857 | 0.333 | 1.000 | 0.975 | 0.300 | 1.000 | 0.958 | 0.600 | <0.001 | 1.000 | 1.000 | 1.000 | 0.800 | 0.200 | NA | 0.667 | 0.200 | 0.500 | 1.000 | 1.000 |
| GLM | 0.500 | 0.929 | 0.667 | 1.000 | 0.950 | 0.700 | 1.000 | 0.946 | 0.667 | <0.001 | 0.750 | 1.000 | 1.000 | 1.000 | 0.600 | NA | 1.000 | 0.333 | 0.500 | 0.909 | 1.000 |
| **Without hepatic fibrosis** | | | |  |  |  |  |  |  |  |  |  |  |  |  |  |  |  |  |  |  |
| DL | 0.857 | 0.967 | 0.889 | 0.911 | 0.994 | 0.931 | 0.974 | 0.998 | 0.898 | 0.889 | 1.000 | 0.833 | 0.800 | 1.000 | 0.833 | 0.889 | 1.000 | 0.750 | 0.800 | 0.923 | 1.000 |
| RF | 0.625 | 1.000 | 1.000 | 0.823 | 1.000 | 1.000 | 0.926 | 1.000 | 1.000 | 0.583 | 1.000 | 1.000 | 0.750 | 1.000 | 1.000 | 0.875 | 1.000 | 1.000 | 0.375 | 1.000 | 1.000 |
| GBM | 0.857 | 0.967 | 0.889 | 0.867 | 1.000 | 0.903 | 0.944 | 1.000 | 0.834 | 0.889 | 0.944 | 1.000 | 0.800 | 1.000 | 0.833 | 0.889 | 1.000 | 0.750 | 0.800 | 0.923 | 1.000 |
| GLM | 0.857 | 0.967 | 0.889 | 0.911 | 0.994 | 0.931 | 0.974 | 0.999 | 0.898 | 0.889 | 0.944 | 1.000 | 0.800 | 1.000 | 0.833 | 0.889 | 1.000 | 0.750 | 0.800 | 0.923 | 1.000 |

AUC, area under the curve for receiver operating characteristic; AUCPR, AUC for precision-recall; DL, deep learning; GBM, gradient boosting machine; GLM, generalized linear model; RF, random forest.

**Supplementary Table 5. Evaluation of** **machine learning models performance in stratified analyses**

|  | RMSE | | | MSE | | | R square | | |
| --- | --- | --- | --- | --- | --- | --- | --- | --- | --- |
|  | Test | Train | Valid | Test | Train | Valid | Test | Train | Valid |
| **Abnormal cerebral function** | | | |  |  |  |  |  |  |
| DL | 0.026 | 0.218 | 0.189 | 0.001 | 0.047 | 0.036 | 0.997 | 0.802 | 0.798 |
| RF | 0.349 | 0.339 | 0.229 | 0.122 | 0.115 | 0.052 | 0.493 | 0.520 | 0.706 |
| GBM | 0.284 | 0.021 | 0.208 | 0.080 | 0.001 | 0.043 | 0.665 | 0.998 | 0.756 |
| GLM | 0.016 | 0.032 | 0.044 | 0.128 | 0.179 | 0.209 | 0.932 | 0.867 | 0.754 |
| **Normal cerebral function** | | | | |  |  |  |  |  |
| DL | 0.421 | 0.306 | 0.283 | 0.178 | 0.093 | 0.080 | 0.290 | 0.593 | 0.668 |
| RF | 0.368 | 0.430 | 0.388 | 0.136 | 0.185 | 0.150 | 0.458 | 0.196 | 0.374 |
| GBM | 0.255 | 0.232 | 0.290 | 0.065 | 0.054 | 0.084 | 0.740 | 0.765 | 0.649 |
| GLM | 0.377 | 0.284 | 0.290 | 0.142 | 0.081 | 0.084 | 0.430 | 0.649 | 0.650 |
| **With hepatic fibrosis** | | | | |  |  |  |  |  |
| DL | 0.499 | 0.397 | 0.466 | 0.249 | 0.158 | 0.217 | 0.005 | 0.228 | -0.560 |
| RF | 0.515 | 0.341 | 0.447 | 0.266 | 0.117 | 0.200 | -0.063 | 0.429 | -0.441 |
| GBM | 0.624 | 0.229 | 0.546 | 0.389 | 0.052 | 0.298 | -0.556 | 0.744 | -1.143 |
| GLM | 0.525 | 0.425 | 0.390 | 0.276 | 0.172 | 0.152 | -0.103 | 0.155 | -0.095 |
| **Without hepatic fibrosis** | | | | |  |  |  |  |  |
| DL | 0.356 | 0.199 | 0.369 | 0.127 | 0.040 | 0.136 | 0.449 | 0.835 | 0.386 |
| RF | 0.459 | 0.356 | 0.228 | 0.211 | 0.127 | 0.052 | -0.123 | 0.484 | 0.767 |
| GBM | 0.408 | 0.144 | 0.400 | 0.167 | 0.021 | 0.160 | 0.274 | 0.914 | 0.280 |
| GLM | 0.356 | 0.199 | 0.369 | 0.127 | 0.039 | 0.136 | 0.449 | 0.386 | 0.835 |

DL, deep learning; GBM, gradient boosting machine; GLM, generalized linear model; RF, random forest; MSE, mean square error; RMSE, root mean square error.

**Supplementary Table 6. Performance evaluation of machine learning**

|  | Brier Score  (95% CI) | Calibration-in-the-large  (95% CI) | Calibration Slope  (95% CI) |
| --- | --- | --- | --- |
| **Overall** |  |  |  |
| DL | 0.141 (0.118, 0.164) | 0.031 (-0.012, 0.074) | 1.081 (0.924, 1.238) |
| RF | 0.159 (0.136, 0.182) | 0.039 (-0.005, 0.083) | 1.025 (0.877, 1.173) |
| GBM | 0.151 (0.127, 0.175) | 0.017 (-0.026, 0.060) | 1.126 (0.960, 1.292) |
| GLM | 0.155 (0.132, 0.178) | 0.024 (-0.019, 0.067) | 1.057 (0.909, 1.205) |
| **Stratified analysis** | |  |  |
| **Normal cerebral function** | |  |  |
| DL | 0.155 (0.110, 0.202) | -0.015 (-0.122, 0.091) | 1.142 (0.792, 1.512) |
| RF | 0.148 (0.105, 0.194) | -0.002 (-0.101, 0.096) | 1.054 (0.745, 1.376) |
| GBM | 0.163 (0.116, 0.213) | -0.023 (-0.131, 0.086) | 1.186 (0.816, 1.576) |
| GLM | 0.161 (0.115, 0.209) | -0.021 (-0.127, 0.085) | 1.167 (0.813, 1.534) |
| **Abnormal cerebral function** | |  |  |
| DL | 0.151 (0.110, 0.193) | -0.018 (-0.106, 0.070) | 1.086 (0.783, 1.397) |
| RF | 0.146 (0.107, 0.187) | -0.004 (-0.085, 0.076) | 1.023 (0.753, 1.300) |
| GBM | 0.163 (0.119, 0.208) | -0.028 (-0.122, 0.066) | 1.153 (0.824, 1.496) |
| GLM | 0.159 (0.117, 0.203) | -0.025 (-0.115, 0.065) | 1.125 (0.813, 1.447) |
| **With hepatic fibrosis** | |  |  |
| DL | 0.163 (0.128, 0.198) | -0.021 (-0.058, 0.016) | 0.920 (0.850, 0.990) |
| RF | 0.157 (0.124, 0.190) | -0.015 (-0.051, 0.021) | 0.940 (0.880, 1.000) |
| GBM | 0.155 (0.122, 0.188) | -0.012 (-0.048, 0.024) | 0.960 (0.900, 1.020) |
| GLM | 0.172 (0.136, 0.208) | 0.035 (-0.003, 0.073) | 0.880 (0.810, 0.950) |
| **Without hepatic fibrosis** | |  |  |
| DL | 0.221 (0.150, 0.300) | 0.158 (-0.144, 0.461) | 0.820 (0.237, 1.474) |
| RF | 0.216 (0.146, 0.297) | 0.094 (-0.171, 0.360) | 0.932 (0.326, 1.570) |
| GBM | 0.224 (0.152, 0.307) | 0.121 (-0.167, 0.409) | 0.871 (0.254, 1.530) |
| GLM | 0.229 (0.155, 0.312) | 0.172 (-0.130, 0.475) | 0.785 (0.215, 1.411) |

CI, confidence interval; DL, deep learning; GBM, gradient boosting machine; GLM, generalized linear model; RF, random forest.

**Supplementary Table 7. Results of 5-fold** **cross validation in WD patients without** **hepatic fibrosis**

|  | Accuracy | AUC | AUC_PR | Precision | Specificity | R^2^ | MSE | RMSE |
| --- | --- | --- | --- | --- | --- | --- | --- | --- |
| **DL** |  |  |  |  |  |  |  |  |
| CV_1 | 1.000 | 1.000 | 1.000 | 1.000 | 1.000 | 0.386 | 0.098 | 0.313 |
| CV_2 | 1.000 | 1.000 | 1.000 | 1.000 | 1.000 | -0.125 | 0.270 | 0.520 |
| CV_3 | 1.000 | 1.000 | 1.000 | 1.000 | 1.000 | 0.094 | 0.170 | 0.412 |
| CV_4 | 0.750 | 0.833 | 0.500 | 0.500 | 0.667 | 0.097 | 0.169 | 0.412 |
| CV_5 | 0.500 | 0.333 | 0.189 | 0.333 | 0.333 | -0.333 | 0.250 | 0.500 |
| **RF** |  |  |  |  |  |  |  |  |
| CV_1 | 1.000 | 1.000 | 1.000 | 1.000 | 1.000 | 0.472 | 0.085 | 0.291 |
| CV_2 | 1.000 | 1.000 | 1.000 | 1.000 | 1.000 | 0.245 | 0.181 | 0.426 |
| CV_3 | 1.000 | 1.000 | 1.000 | 1.000 | 1.000 | 0.466 | 0.100 | 0.316 |
| CV_4 | 0.750 | 0.833 | 0.500 | 0.500 | 0.667 | 0.111 | 0.167 | 0.408 |
| CV_5 | 0.500 | 0.333 | 0.189 | 0.333 | 0.333 | -0.423 | 0.267 | 0.517 |
| **GBM** |  |  |  |  |  |  |  |  |
| CV_1 | 1.000 | 1.000 | 1.000 | 1.000 | 1.000 | 0.704 | 0.047 | 0.218 |
| CV_2 | 0.800 | 0.667 | 0.451 | 0.667 | 0.667 | -0.353 | 0.325 | 0.570 |
| CV_3 | 1.000 | 1.000 | 1.000 | 1.000 | 1.000 | 0.640 | 0.067 | 0.260 |
| CV_4 | 0.750 | 0.833 | 0.500 | 0.500 | 0.667 | 0.094 | 0.170 | 0.412 |
| CV_5 | 0.500 | 0.333 | 0.189 | 0.333 | 0.333 | -1.215 | 0.415 | 0.644 |
| **GLM** |  |  |  |  |  |  |  |  |
| CV_1 | 1.000 | 1.000 | 1.000 | 1.000 | 1.000 | 0.527 | 0.076 | 0.275 |
| CV_2 | 1.000 | 1.000 | 1.000 | 1.000 | 1.000 | 0.073 | 0.222 | 0.472 |
| CV_3 | 1.000 | 1.000 | 1.000 | 1.000 | 1.000 | 0.674 | 0.061 | 0.247 |
| CV_4 | 0.750 | 0.833 | 0.500 | 0.500 | 0.667 | 0.226 | 0.145 | 0.381 |
| CV_5 | 0.500 | 0.333 | 0.189 | 0.333 | 0.333 | -0.008 | 0.189 | 0.435 |

AUC, area under the curve; AUC_PR, AUC for precision-recall; CV, cross validation; DL, deep learning; GBM, gradient boosting machine; GLM, generalized linear model; RF, random forest; MSE, mean square error; RMSE, root mean square error.
